# Role of the clock gene Bmal1 and the gastric ghrelin-secreting cell in the circadian regulation of the ghrelin-GOAT system

**DOI:** 10.1038/srep16748

**Published:** 2015-11-18

**Authors:** J. Laermans, L. Vancleef, J. Tack, I. Depoortere

**Affiliations:** 1 Gut Peptide Research Lab, Translational Research Center for Gastrointestinal Disorders (TARGID), University of Leuven, Leuven, Belgium

## Abstract

As adequate food intake is crucial to survival, organisms have evolved endogenous circadian clocks to generate optimal temporal patterns of food-related behavior and physiology. The gastric ghrelin-secreting cell is thought to be part of this network of peripheral food-entrainable oscillators (FEOs), regulating the circadian release of this orexigenic peptide. This study aimed to determine the role of the core clock gene Bmal1 and the gastric ghrelin-secreting cell as an FEO in the circadian rhythmicity of ghrelin expression and secretion *in vivo* and *in vitro*. Bmal1-deficient mice not only lacked circadian rhythmicity in plasma ghrelin levels and food intake, but also showed decreased gastric mRNA expression of ghrelin and ghrelin O-acyltransferase (GOAT), the ghrelin activating enzyme. Furthermore, in the absence of the hypothalamic master clock, food-related stimuli entrained the molecular clock of gastric ghrelinoma cells to regulate the rhythmic release of ghrelin. Divergent responses in octanoyl and total ghrelin release towards different food cues were observed, suggesting that the FEO also regulates the circadian rhythmicity of GOAT. Collectively, these findings indicate that circadian rhythmicity of ghrelin signaling requires Bmal1 and is driven by a food-responsive clock in the gastric ghrelin-secreting cell that not only regulates ghrelin, but also GOAT activity.

Ghrelin, a 28-amino acid gut peptide, was identified as the endogenous ligand of the growth hormone secretagogue receptor type 1A in 1999^1^. The posttranslational octanoylation of ghrelin at Serine-3, catalyzed by the enzyme ghrelin O-acyltransferase (GOAT), was reported to be indispensable for its biological activity^2^,^3^. Nevertheless, octanoyl ghrelin only represents about 10% of all circulating ghrelin, whereas the remaining 90% is non-acylated^4^. During the years following its discovery, it was shown that ghrelin not only mediates growth hormone release, but also governs other essential physiological functions. Ghrelin increases food intake by stimulating the release of the orexigenic neuropeptides agouti-related peptide (AgRP) and neuropeptide Y (NPY) from the hypothalamic arcuate nucleus^5^ and is therefore considered to act as a physiological meal initiator. Furthermore, it exerts stimulatory effects on adiposity and gastric motility and represents an important mediator of glucose homeostasis^6^,^7^. Other physiological functions of ghrelin include modulation of cell proliferation, the immune system, cartilage and bone homeostasis, sleep, memory and behavioral influences.

As a consequence of the combined action of several mediators, plasma ghrelin levels continuously fluctuate throughout the course of the day. Nutritional status represents a first and well-documented regulator of endogenous ghrelin release. In rodents, circulating ghrelin levels are increased by fasting^8^,^9^ and suppressed by refeeding^9^. Similarly, plasma ghrelin levels in healthy volunteers increase approximately one hour before a regularly timed meal and decrease sharply afterwards^10^. The fasting-induced increase in plasma ghrelin levels relies on the cholinergic and adrenergic arms of the autonomic nervous system^11^,^12^,^13^, whereas the postprandial suppression of ghrelin levels seems to be mediated by the caloric content and type of ingested nutrients^14^,^15^. Although incompletely understood, nutrient sensing seems predominantly mediated by activation of different taste receptors present on the enteroendocrine cells, including the gastric ghrelin cells^16^,^17^,^18^,^19^.

In addition to nutritional status, the circadian system also plays a pivotal role in determining plasma ghrelin levels, by fine-tuning the timing of ghrelin release throughout the 24-hour cycle. Plasma ghrelin levels display a circadian rhythm in rodents and humans, with peak and trough levels being reached during the inactive and active phase, respectively^20^,^21^,^22^,^23^. The circadian system relies on the orchestrated expression of a set of clock genes (Clock, Bmal1, Per1-3, Cry1-2) in nearly every cell in the body, which participate in different interlocked transcriptional-translational feedback loops^24^. The near 24-hour rhythm is imposed onto the transcription of numerous clock-controlled genes and translated into circadian rhythmicity of numerous molecular and physiological processes. In general, mammalian timekeeping depends on a hypothalamic central pacemaker in the suprachiasmatic nuclei (SCN), which receives environmental input from the light/dark-cycle and sets the time for all other peripheral clocks via neural and humoral signaling^24^. Besides light, food also constitutes a very potent signal for the synchronization of peripheral clocks. Feeding nocturnal rodents exclusively during daytime inverses the phase of peripheral clock gene expression, realigning it with the rhythm of food availability^25^,^26^. Thus, the presence of food can overrule the entraining signals from the SCN and become the dominant synchronizing signal, resulting in uncoupling between the peripheral and master clocks. This peripheral clock entrainment by feeding cues persists in SCN-lesioned mice^27^, implying that the circadian rhythm of feeding is mediated by separate food-entrainable oscillators (FEOs). As ghrelin and the clock genes Per1 and Per2 were rhythmically co-expressed within the gastric ghrelin cells and the phase of this rhythm was controlled by the time of food availability, it was postulated that FEOs might be located within the ghrelin-secreting cells of the stomach^28^.

This study aimed to explore the importance of clock gene function in the circadian rhythmicity of ghrelin expression and secretion. First of all, we investigated the effect of genetic deletion of the core clock gene Bmal1 on the 24-hour fluctuation in plasma ghrelin levels and gastric ghrelin and GOAT expression in mice. Secondly, we investigated the possibility that ghrelin-secreting cells might act as FEOs by studying the effect of fasting or feeding cues as triggers for circadian rhythmicity in a ghrelinoma cell line.

## Results

### Circadian regulation of the ghrelin-GOAT system in mice

**Figure 1:**
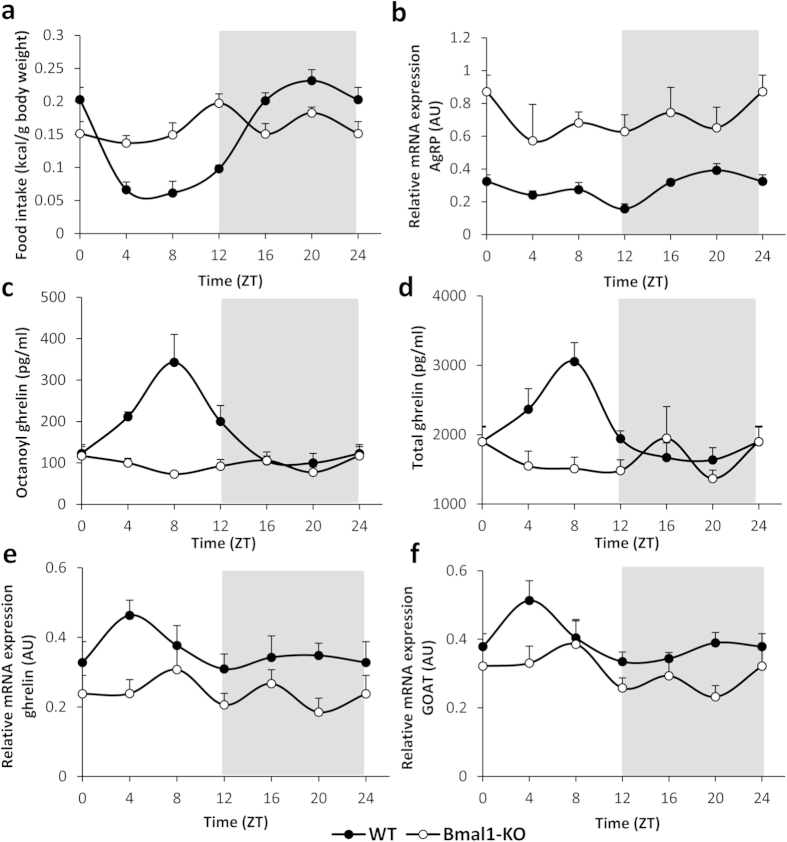
Genetic deletion of Bmal1 abolishes circadian rhythmicity and alters food intake, ghrelin release and expression. (**a**) Food intake, (**b**) hypothalamic relative mRNA expression levels of AgRP, (**c**) plasma octanoyl ghrelin levels and (**d**) plasma total ghrelin levels in *ad libitum*-fed WT or Bmal1-KO mice. (**e**,**f**) Relative mRNA expression levels of ghrelin (**e**) and GOAT (**f**) in the stomach of WT or Bmal1-KO mice (n = 7–8 mice/time point/genotype). Light and dark phases are shaded in white and gray, respectively. (AU = arbitrary unit).

WT mice displayed typical nocturnal behavior, consuming the majority of their food during the dark-cycle (Fig. 1a). Food intake started to increase from *zeitgeber* time (ZT) 8 on and reached peak levels, which determine the acrophase of the rhythm, at ZT 21. Because food intake is stimulated by the release of the orexigenic agouti-related peptide (AgRP) from the arcuate nucleus, we determined the hypothalamic mRNA expression levels of this neuropeptide. The acrophase (ZT 21) of the circadian rhythm of AgRP mRNA expression (period of 23 hours, *P* < 0.001) coincided with the acrophase of food intake (Fig. 1b). In contrast to their WT counterparts, the circadian feeding rhythm was absent in Bmal1-KO mice. Moreover, when correcting for their significantly lower body weight (Bmal1-KO vs. WT: 21.13 ± 0.40 g vs. 24.67 ± 0.44 g; *P* < 0.001), they tended (*P* = 0.07) to eat more calories over the 24-hour period compared to WT mice (Bmal1-KO vs. WT: 0.97 vs. 0.86 kcal/g body weight) (Fig. 1a). In addition, mean hypothalamic AgRP mRNA expression levels were increased (*P* < 0.001) compared to WT mice and mRNA accumulation was arrhythmic in Bmal1-KO mice (Fig. 1b).

In WT mice, plasma octanoyl ghrelin levels oscillated in a circadian manner (period of 23 hours; *P* < 0.001) (Fig. 1c), reaching their acrophase at ZT 8. Similar results were obtained for plasma total ghrelin levels (period of 23 hours, *P* < 0.001; acrophase at ZT 7) (Fig. 1d). In Bmal1-KO mice, plasma octanoyl ghrelin levels displayed an ultradian (biphasic, i.e. with a period of 12 hours, *P* < 0.05; acrophase at ZT 2) rhythm, whereas total ghrelin levels were arrhythmic (Fig. 1c,d).

**Figure 2:**
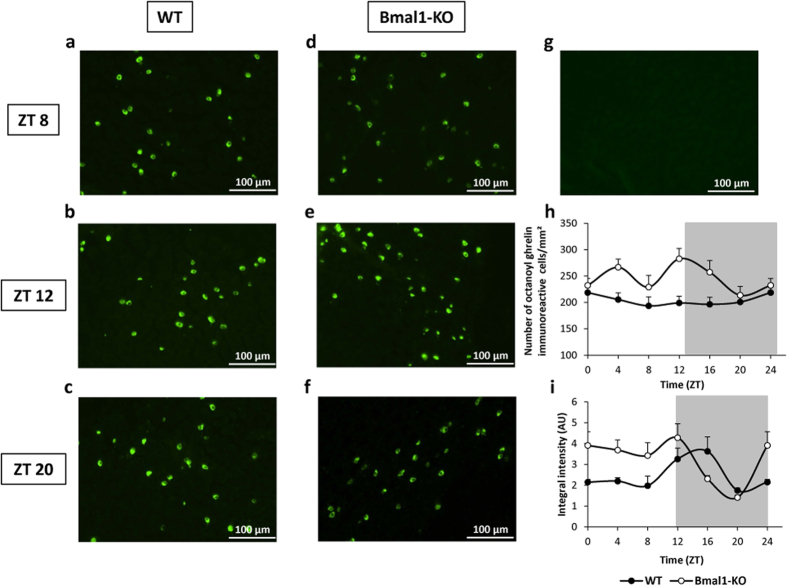
Genetic deletion of Bmal1 abolishes circadian rhythmicity in gastric octanoyl ghrelin content. (**a–f**) Representative pictures of corpus sections stained for octanoyl ghrelin of WT (**a–c**) or Bmal1-KO (**d–f**) mice at ZT 8 (**a,d**), ZT 12 (**b,e**) and ZT 20 (**c,f**). (**g**) Picture of a no primary antibody negative control section. (**h**) Number of octanoyl ghrelin immunoreactive cells/mm^2^ and (**i**) intensity of the octanoyl ghrelin staining in sections of the corpus of WT or Bmal1-KO mice (n = 3 mice/time point/genotype). Light and dark phases are shaded in white and gray, respectively. (AU = arbitrary unit).

To clarify whether the rhythmicity in circulating ghrelin was also reflected in changes in gastric ghrelin content, immunofluorescence staining for ghrelin was performed on stomach tissue sections of WT and Bmal1-KO mice. Figure 2 shows representative pictures of the gastric ghrelin staining at ZT 8, 12 and 20, including a negative control. Quantitative analysis revealed that in WT mice, the number of gastric octanoyl ghrelin immunoreactive cells did not change throughout the 24 h-cycle (Fig. 2a–c,h). Interestingly, Bmal1-KO mice presented with an overall increased number of octanoyl ghrelin immunoreactive cells compared to WT mice (genotype *P* < 0.001), which oscillated in an ultradian fashion (period of 12 hours, *P* < 0.05) (Fig. 2d–f,h). In WT mice, the intensity of the octanoyl ghrelin staining oscillated in a circadian fashion (period of 20 hours, *P* < 0.01; acrophase at ZT 14) (Fig. 2i). Bmal1-KO mice showed a significant (*P* < 0.001) increase in the mean intensity of the octanoyl ghrelin staining, as well as a shift in the circadian rhythm compared to WT mice (period of 24 hours, *P* < 0.01; acrophase at ZT 6) (Fig. 2i).

Circadian oscillations in gastric mRNA expression levels of ghrelin (period of 20 hours, *P* < 0.05) and GOAT, the enzyme responsible for ghrelin’s octanoylation (period of 23 hours, *P* < 0.05) were also observed in WT mice (Fig. 1e,f). The acrophase of these rhythms occurred at ZT 5 and ZT 4 for ghrelin and GOAT mRNA expression, respectively. Loss of Bmal1 also led to a decrease in mean gastric mRNA expression of ghrelin compared to WT mice (Bmal1-KO vs. WT: 0.23 vs. 0.33 arbitrary units; *P* < 0.001) and resulted in an ultradian rhythm (period of 10 hours, *P* < 0.01; acrophase at ZT 6) (Fig. 1e). Similarly, average mRNA expression levels of GOAT were decreased in Bmal1-KO mice compared to WT mice (Bmal1-KO vs. WT: 0.29 vs. 0.39 AU; *P* < 0.001) and levels were arrhythmic (Fig. 1f).

### Circadian regulation of the ghrelin-GOAT system in a mouse ghrelinoma cell line

**Figure 3:**
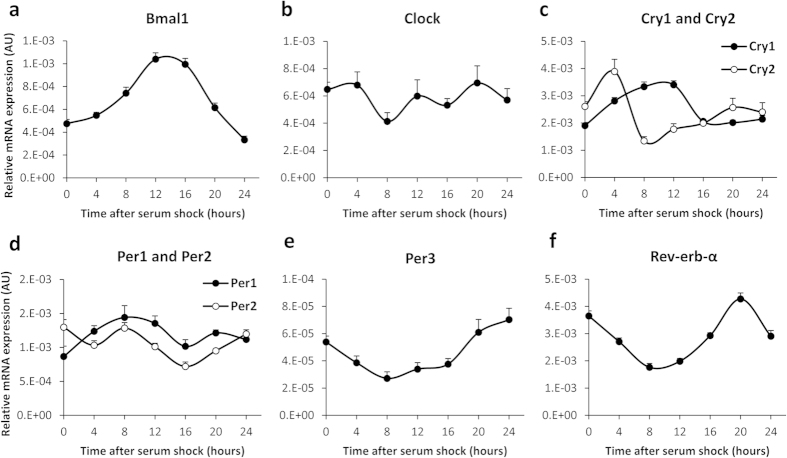
Serum-shocked MGN3-1 ghrelinoma cells exhibit robust circadian rhythmicity in clock gene mRNA accumulation. Relative mRNA expression levels of (**a**) Bmal1, (**b**) Clock, (**c**) Cry1 and Cry2, (**d**) Per1 and Per2, (**e**) Per3 and (**f**) Rev-erb-α in MGN3-1 ghrelinoma cells in response to serum shock synchronization. (AU = arbitrary unit).

To unravel whether the gastric ghrelin-secreting cell itself can act as a FEO, we made use of the murine MGN3-1 gastric ghrelinoma cell line. Since these cultured cells no longer receive input from the master pacemaker, the cellular clock genes cycle with a similar period, but a different phase. Synchronization of the circadian cycles by incubating the cells with high concentrations of serum for 2 hours^29^ induced robust oscillations in mRNA transcript accumulation of all clock genes, except for Clock (Fig. 3).

**Figure 4:**
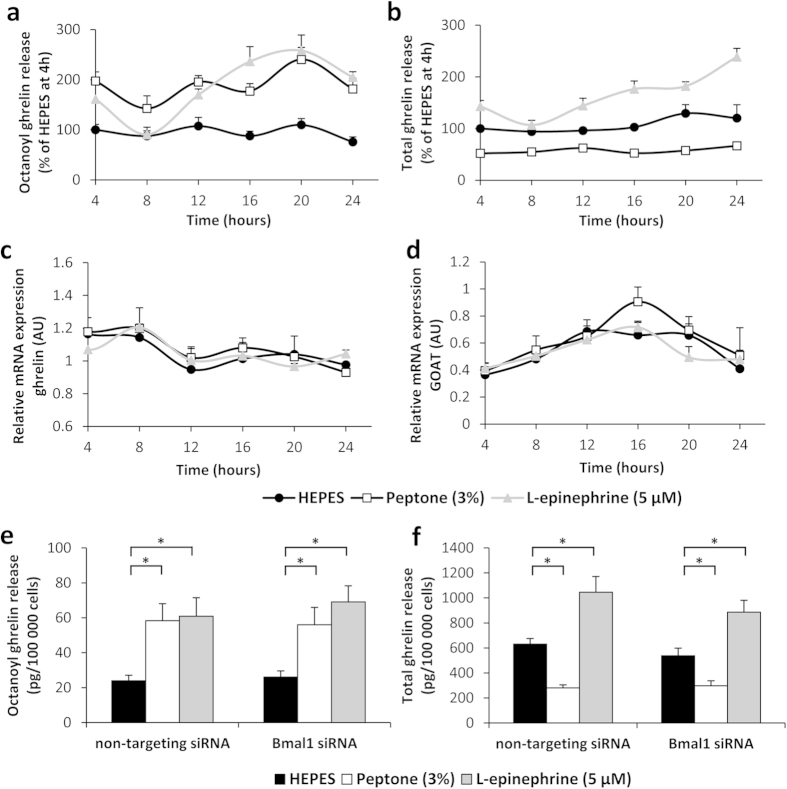
Food-related cues induce circadian rhythmicity in ghrelin release by MGN3-1 ghrelinoma cells. (**a**) Octanoyl ghrelin release, (**b**) total ghrelin release and relative mRNA expression levels of (**c**) ghrelin and (**d**) GOAT of serum shocked MGN3-1 cells after a 3-hour stimulation with vehicle (HEPES), peptone (3%) or L-epinephrine (5 μM) at 4-hour intervals (N = 3 independent experiments; n = 14 wells/stimulus/time point). (**e**) Octanoyl ghrelin release and (**f**) total ghrelin release of MGN3-1 cells transfected with Bmal1 siRNA or a non-targeting siRNA pool as negative control, after a 3-hour stimulation with vehicle (HEPES), peptone (3%) or L-epinephrine (5 μM) (N = 2 independent experiments; n = 8 wells/stimulus). *P < 0.001 vs. HEPES. (AU = arbitrary unit).

Next, we investigated whether ghrelin secretion follows a circadian rhythm in response to stimulation with compounds that mediate ghrelin release either during the fasting (L-epinephrine) or fed state (peptone, a mixture of amino acids and peptides). To this end, serum shocked cells were incubated with these compounds every 4 hours for a period of 3 hours. Basal octanoyl ghrelin secretion (vehicle treatment) did not show circadian rhythmicity, in contrast to total ghrelin secretion (period of 26 hours, *P* < 0.01; acrophase at ZT 22) (Fig. 4a,b). Stimulation with peptone triggered rhythmicity in octanoyl (period of 21 hours, *P* < 0.05; acrophase at ZT 19), but not total ghrelin secretion (Fig. 4a,b). L-epinephrine elicited robust circadian rhythmicity in both octanoyl (period of 20 hours, *P* < 0.001; acrophase at ZT 18) and total ghrelin release (period of 26 hours, *P* < 0.001; acrophase at ZT 21)(Fig. 4a,b).

Moreover, octanoyl ghrelin release by the MGN3-1 cells was significantly increased during incubation with peptone (*P* < 0.001) or L-epinephrine (*P* < 0.001), regardless of time (Fig. 4a). In contrast, total ghrelin release was inhibited by peptone (*P* < 0.001), but stimulated by incubation with L-epinephrine (*P* < 0.001) (Fig. 4b). Ghrelin mRNA expression levels in the MGN3-1 cells did not show rhythmicity and were not significantly altered by treatment (Fig. 4c). In contrast, GOAT mRNA expression levels did display circadian rhythmicity, but were also not affected by treatment (for all three compounds: period of 24 hours, P < 0.01) (Fig. 4d).

To elucidate the role of Bmal1 in the circadian rhythmicity of the secretory ghrelin responses towards peptone and L-epinephrine, RNA silencing for Bmal1 was performed. Transfection with Bmal1 siRNA resulted in 57 ± 3% knockdown of Bmal1 mRNA expression at 16 hours. Two-way ANOVA analysis revealed that peptone stimulated octanoyl ghrelin and inhibited total ghrelin secretion to a similar extent in cells transfected with either Bmal1 siRNA or non-targeting siRNA (Fig. 4e,f). In addition, the stimulatory effect of L-epinephrine on octanoyl and total ghrelin secretion persisted after knockdown of Bmal1 (Fig. 4e,f).

## Discussion

Ever since clock genes were described to be expressed outside the SCN, it became clear that peripheral clocks are not only entrained by hormonal and neuronal signals from the central pacemaker. Food and food-related cues quickly emerged as very potent synchronizers of peripheral clock gene expression, even in SCN-lesioned mice^25^,^27^. Therefore, the increases in arousal and activity in anticipation of a regularly scheduled meal are believed to depend on a separate food-entrainable oscillator (FEO). Since plasma ghrelin levels not only surge prior to mealtime, but are also controlled by the circadian system, this orexigenic peptide imposed itself as a prime candidate output signal of the FEO, that would be able to provide important metabolic input to the circadian system. The hypothesis was strengthened by the findings that exogenous administration of ghrelin also stimulates activity in the absence of food deprivation, whereas elimination of its receptor seems to attenuate food anticipatory locomotor activity^28^,^30^. Nevertheless, the mechanisms underlying the circadian control of ghrelin production and/or secretion have remained unclear.

In this study, we are the first to demonstrate that the genetic deletion of the core clock gene Bmal1 not only abolishes the circadian rhythmicity of ghrelin signaling and hence the circadian rhythm of food intake, but also attenuates the absolute mRNA production of ghrelin and GOAT. In addition, we provide convincing evidence that in the absence of the master clock, food-related cues can entrain the molecular clock in the gastric ghrelin-secreting cell to regulate the rhythmic secretion of ghrelin. Moreover, the diverging responses in octanoyl and total ghrelin release towards the food cue peptone, together with the rhythmic expression of GOAT but not of ghrelin mRNA in the ghrelinoma cells, suggest that the ghrelin cell as FEO not only regulates the circadian rhythmicity of ghrelin but perhaps more importantly of GOAT.

Our *in vivo* study in WT mice confirms the previous observations that plasma ghrelin levels oscillate in a circadian way and reach peak levels during the inactive phase of the organism^20^,^21^,^22^,^23^. The increase in ghrelin and GOAT mRNA accumulation at ZT 4 suggests that the circadian system anticipated the release of ghrelin at ZT 8, probably conveying rhythmicity to these genes via the E-box elements in their promoter regions^31^. The number of immunoreactive cells remained constant over the 24-hour cycle, indicating that under normal circumstances, all gastric ghrelin cells equally contribute to the secretion of this peptide. Furthermore, the intensity of the ghrelin staining ran in antiphase with the plasma ghrelin levels, highlighting that the stomach serves as a major source of circulating ghrelin^32^,^33^,^34^.

Bmal1 deficiency was previously shown to interfere with circadian rhythm generation, both at the behavioral (locomotor activity) and molecular level (mRNA expression levels of other clock genes in the SCN and transcriptional output genes in the liver)^35^,^36^. Consistent with these results, we demonstrated that genetic deletion of Bmal1 resulted in abolished circadian rhythmicity of both octanoyl and total ghrelin plasma levels and hence of the ghrelin-GOAT system. At the functional level, this circadian disruption of the ghrelin-GOAT system might contribute to the observed disturbances in the rhythmicity of the eating pattern of the Bmal1-KO mice. The finding that these mice switched their typical nocturnal feeding behavior to a constant consumption of food during the light/dark-cycle was also reflected in a loss of rhythmicity in the expression of the hypothalamic neuropeptide AgRP. However, this loss of ghrelin rhythmicity does not lie at the basis of the tendency towards an overall increase in calorie intake in the Bmal1-KO mice. One has to bear in mind that the control of food intake involves a complex interplay between different organs (including the gut, brain and adipose tissue) that use a variety of orexigenic and anorexigenic peptides and hormones. We speculate that the genetic deletion of Bmal1 does not only affect ghrelin, but also induces alterations in the levels of these other (an)orexigenic signals, ultimately resulting in an overall increase in calorie intake in the Bmal1-KO mice.

Despite their tendency towards increased calorie intake, Bmal1-KO mice exhibited significantly lower body weight compared to WT controls. Several studies have highlighted that deletion of circadian clock genes in mice can drastically alter body weight and body composition^37^,^38^,^39^,^40^. Bmal1 was reported to play a crucial role in the control of adipogenesis and lipid metabolism^41^,^42^. The reduced fat storing capacity of the Bmal1-KO mice may explain why the increase in food intake was not accompanied by an increase in body weight.

Recently, global loss of Bmal1 was shown to induce decreased insulin, but increased adiponectin and leptin plasma levels, which might serve as a compensatory effort to maintain metabolic homeostasis^43^. In a similar way, the decrease in plasma ghrelin levels in Bmal1-KO mice might be utilized to counteract their tendency towards elevated food intake, which may be driven or exacerbated by the dysregulation of AgRP. Another study showed that Bmal1-KO mice displayed significantly lower plasma corticosterone levels compared to age-matched WT controls^44^. This hypocortisolism was not accompanied by alterations in ACTH levels or developmental abnormalities of the adrenal gland, but involved adrenal ACTH resistance induced by disruption of normal adrenal clock function. In this study, we observed that loss of Bmal1 resulted in significantly less accumulation of ghrelin and GOAT mRNA transcripts in the stomach. Turek *et al.* previously reported a similar decrease in the absolute mRNA expression levels of the energy-regulatory peptides ghrelin and orexin in the mediobasal hypothalamus of *Clock* mutant mice^40^. These findings strengthen the idea that the circadian system not only regulates the timing of gene transcription, but also the absolute expression levels of different mRNA transcripts in both the brain and periphery. Moreover, it seems that the overall decrease in ghrelin and GOAT mRNA expression in Bmal1-KO mice might be the consequence of impaired ghrelin secretion. Indeed, the increased number and intensity of the octanoyl ghrelin immunoreactive cells in the corpus of the Bmal1-KO mice might reflect their incapability to secrete the peptide from the stomach, hence making it unnecessary to increase ghrelin mRNA expression.

Although our *in vivo* study demonstrated near 24-hour changes in the ghrelin-GOAT system, this rhythmicity might have been conveyed from the SCN to the periphery and therefore be entrained by the exogenous photic information of the light/dark-cycle. Moreover, other indirect mechanisms (*e.g.* leptin rhythmicity^20^) might have elicited the observed ghrelin-GOAT patterns. In an effort to rule out these possibilities and establish whether the gastric ghrelin-secreting cells themselves display an endogenous circadian response to feeding-related cues, serum shocked MGN3-1 ghrelinoma cells were subjected to stimuli that mimicked the fed (peptone) or fasted (L-epinephrine) state at different time points. Since it is well established that the activity of the sympathetic nervous system exhibits diurnal variations^45^,^46^,^47^ and that adrenergic signaling can directly increase ghrelin release by binding to β1-adrenergic receptors located on the ghrelin-secreting cells^48^, it is not surprising that the secretory response of the MGN3-1 cells towards adrenergic stimulation (L-epinephrine) displayed a circadian pattern. These results indicate that the previously reported role for peripheral catecholamines as one of the entraining signals for the FEO in the rodent liver^49^,^50^ also holds true for the murine stomach. Our finding that circadian rhythmicity of octanoyl ghrelin secretion can be induced after stimulation with peptone, but not HEPES, reinforces the hypothesis that the gastric ghrelin-secreting cell might act as a food-entrainable oscillator. Similar to our results, Gil-Lozano *et al.* reported a circadian rhythm in the secretion of GLP-1 by the rodent L-cell in response to different secretagogues^51^. The observed circadian patterns in the secretory responses of these gut peptide-containing cells indicate a tight coordination between intestinal function, nutrient ingestion and the circadian system.

Interestingly, unlike in our *in vivo* study, ghrelin mRNA expression did not oscillate in a circadian fashion in the MGN3-1 cells, indicating that external input was required for the rhythm observed in ghrelin mRNA levels *in vivo*. In contrast to ghrelin, GOAT mRNA expression did show a circadian pattern in the cultured cells, indicating that the rhythmicity of this enzyme is regulated independently from the master clock.

The interpretation of the results was complicated by the observation that the pattern of total ghrelin secretion in response to the used stimuli did not always match that of octanoyl ghrelin. While HEPES buffer did not induce rhythmicity in octanoyl ghrelin secretion, it did so in total ghrelin secretion. Quite the opposite was observed for peptone, with rhythmicity being detected in octanoyl, but not in total ghrelin secretion. In addition, L-epinephrine was capable of inducing rhythmicity in both ghrelin forms. These differences were not accompanied by alterations in the mRNA expression patterns of ghrelin or GOAT. Nevertheless, we cannot exclude that these feeding or fasting cues triggered changes at the protein level or affected GOAT enzyme activity. Future studies should further investigate whether the circadian control of ghrelin signaling might indeed be mediated via temporal differences in the activity or protein levels of this unique octanoylating enzyme.

Collectively, these results indicate that the molecular clock in the gastric ghrelin-secreting cells regulates not only ghrelin, but also GOAT activity, in response to different food-related stimuli. Moreover, they suggest that both during the fasted and fed state, the regulation of ghrelin secretion is even more complex than previously thought. Further studies are required to further unravel the underlying mechanisms.

Transfection of the ghrelinoma cells with siRNA specific for Bmal1 did not alter the secretory response of the ghrelin-secreting cells towards peptone or L-epinephrine. Given that both stimuli were capable of inducing a circadian pattern in the secretory response of the MGN3-1 cells and that Bmal1-KO mice lacked circadian rhythmicity in ghrelin release, this was a rather counterintuitive finding. However, siRNA-mediated knockdown of Bmal1 might have been counteracted by the serum shock procedure. The high concentrations of serum needed to synchronize the cellular circadian clocks may have stimulated cell growth and division, thereby interfering with the knockdown procedure. On the other hand, recent studies suggest that the clock network utilizes active compensatory mechanisms rather than simple redundancy to act as a genetic buffering system to maintain clock function when facing genetic and environmental perturbation^52^,^53^. The knockdown of Bmal1 could therefore be counteracted by other components of the circadian system, thereby ensuring the resilience of the molecular clock.

In conclusion, this study highlights the importance of the core clock gene Bmal1 in the circadian rhythmicity of ghrelin and hence in the circadian rhythmicity of food intake. The finding that the gastric ghrelin-secreting cell exhibits an endogenous circadian secretory response towards food-related cues emphasizes the importance of the timing of food consumption in determining ghrelin release. Furthermore, as the circadian system also seems to exert its control of ghrelin signaling through the octanoylating enzyme GOAT, these results stress the need for the further characterization of the role of this unique enzyme and the development of pharmacological modulators of GOAT activity.

## Methods

### Animals

Breeding couples of mice heterozygous for Bmal1 were kindly provided by R. Lijnen (KU Leuven, Leuven, Belgium). All KO mice and their WT littermates were bred in the animal facility of the KU Leuven and genotyped by PCR analysis performed on total genomic DNA from the tail. Mice were housed in a temperature-controlled environment (20–22 °C) under a 12-h/12-h light/dark-cycle (where ZT 0 is lights-on by convention) and had *ad libitum* access to chow and drinking water. All experiments were approved by the Ethical committee for Animal Experiments of the KU Leuven and carried out in accordance with the approved guidelines.

### Experimental design: circadian regulation of ghrelin in WT and Bmal1-KO mice

WT and Bmal1-KO mice (50% male and 50% female) between 12 and 16 weeks of age were sacrificed over the course of 24 hours at 4-hour intervals. Blood was taken by cardiac puncture and processed for ghrelin determination. The hypothalamus was harvested, stored in RNAlater (Qiagen, Hilden, Germany) and processed for real-time PCR. The stomach was cut open along the greater curvature. A strip was cut vertically from the top to the bottom of the central part of the stomach, stored in RNAlater and processed for real-time PCR. The left part of the stomach was immediately fixed for immunohistochemistry.

### Cell culture, serum shock procedure and stimulation experiments

MGN3-1 ghrelinoma cells^54^ were cultured in DMEM supplemented with 10% fetal bovine serum (FBS) and a mixture of 1% penicillin (100 U/ml) and streptomycin (100 mg/ml) (P/S) at 37 °C in 5% CO_2_. Cells were synchronized using the following serum shock procedure. One day after seeding, the medium was exchanged with serum-free DMEM. After twelve hours, this was replaced by serum-rich medium (DMEM + P/S, supplemented with 50% horse serum) for 2 hours. Afterwards, cells were put on DMEM + 2% FBS until the time point of stimulation. At six different time points throughout the 24-hour cycle, synchronized cells were incubated for 3 hours in vehicle (HEPES buffer), 3% peptone (an enzymatic digest of casein, Sigma (St. Louis, MO, USA) or 5 μM L-epinephrine (Sigma). The cell supernatant was processed for ghrelin radioimmunoassay (RIA) as described below. Cells were processed for real-time PCR.

### Small-interfering RNA experiments

A mixture of four predesigned siRNAs targeting the sequence of Bmal1 was purchased from GE Healthcare. To detect off-target effects, a mixture of four siRNA constructs designed to target no known genes in human, mouse or rat (Non-Targeting siRNA Pool, GE Healthcare) was used as a negative control siRNA. Transfection of ghrelinoma cells with siRNA (25 nM) was accomplished with Interferin (Polyplus transfection). After 48 hours, cells were subjected to serum shock synchronization (as described above) and put on DMEM + 2% FBS for 16 hours. Then, the synchronized cells were incubated for 3 hours in vehicle (HEPES buffer), 3% peptone or 5 μM L-epinephrine. Transfection efficiency was determined by real-time PCR. The cell supernatant was processed for ghrelin RIA.

### Ghrelin RIA

Cell supernatants from ghrelinoma cells or plasma samples were acidified (10% HCl), extracted on a Sep-Pak C18 column and vacuum-dried. The assay for octanoyl ghrelin was performed as previously described^19^ using the tracer ^125^I[Tyr^24^] human ghrelin [1–23] and a rabbit antibody against human ghrelin [1–8], which does not recognize non-acylated ghrelin. For determination of total ghrelin, a rabbit antibody raised against human ghrelin [14–28], which recognizes both octanoyl and non-acylated ghrelin, was used.

### Quantitative real-time PCR

Total RNA was isolated using the Qiagen RNeasy Mini Kit (Qiagen, Hilden, Germany) and reverse transcribed to cDNA using SuperScript II Reverse Transcriptase (Invitrogen, Carlsbad, CA, USA). Quantitative real-time PCR was performed as previously described^55^. Primer sequences are shown in Table 1. Relative expression levels were expressed relative to GAPDH and corrected for inter-run variability.

### Immunohistochemistry

Stomach tissues (3 mice/genotype/time point) were fixed with 4% paraformaldehyde for 2 h (4 °C) followed by cryoprotection in 30% sucrose at 4 °C overnight. Cryostat sections (6 μm) were incubated for 2 h in 0.1 M PBS containing 10% donkey serum and 0.3% Triton X-100 and then incubated with rabbit anti-octanoyl ghrelin (Ab5044, in-house developed) at 4 °C overnight. A no primary antibody control was included to test for non-specific binding. After washing, the sections were incubated with donkey anti-rabbit Alexa488 (Santa Cruz Biotechnology) for 2 h at room temperature. Sections were mounted in Citifluor and visualized under a fluorescence microscope (Olympus BX41). Of each stomach, two sections were analyzed using Cell^F Imaging Software (Olympus Soft Imaging Solutions GmbH, Münster, Germany). The number of ghrelin-positive cells was counted in 4 randomly chosen fields (20×). The intensity of the staining was calculated and expressed as a gray-value between 0 (darkest pixel) and 0.65535 (brightest pixel).

### Statistical analysis

Data are presented as means ± SEM. Circadian rhythm analysis was performed using the free Cosinor software (version 2.3), which calculates the circadian period of a data set using the cosinor procedure as described by Nelson *et al.* in 1979^56^. All other data were analyzed using Statistica 11 (StatSoft Inc., Tulsa, OK, USA). Student’s t-test was performed to detect differences between two independent groups, whereas one- or two-way ANOVA was used to compare more groups. Significance was accepted at the 5% level.

## Additional Information

**How to cite this article**: Laermans, J. *et al.* Role of the clock gene Bmal1 and the gastric ghrelin-secreting cell in the circadian regulation of the ghrelin-GOAT system. *Sci. Rep.* **5**, 16748; doi: 10.1038/srep16748 (2015).

## Figures and Tables

**Table 1:** Forward and reverse primer sequences.

Gene	Forward primer	Reverse primer
GAPDH	CCCCAATgTgTCCgTCgTg	gCCTgCTTCACCACCTTCT
Ghrelin	CCAgAggACAgAggACAAgC	ACATCgAAgggAgCATTgAA
GOAT	ATTTgTgAAgggAAggTggAg	CAggAgAgCAgggAAAAAgAg
Bmal1	CgTTTCTCgACACgCAATAgAT	TCCTgTggTAgATACgCCAAAA
Clock	TCTACAgAAgAgCATTgATTTTTTgC	TCATTACTAAggAATgTgggTTTCC
Cry1	CCCTgTgggTTTTggTAggA	TgCAgggAAgCCTCTTAggA
Cry2	ggCgTggAggTggTgACT	TggTTTCTgCCCATTCAgTTC
Per1	CCgAATACACACTTCgAAACCAg	TCCCgTTTgCAACgCAg
Per2	gATgACAgAggCAgAgCACAAC	TTTgTgTgCgTCAgCTTTgg
Per3	ggTCCTCTgCAgggCACTT	TTCAgACATTCTgTTTCggTCTTC
Rev-erb-α	CCCTggACTCCAATAACAACACA	gCCATTggAgCTgTCACTgTAg
